# Human Polyclonal Antibodies Produced through DNA Vaccination of Transchromosomal Cattle Provide Mice with Post-Exposure Protection against Lethal Zaire and Sudan Ebolaviruses

**DOI:** 10.1371/journal.pone.0137786

**Published:** 2015-09-30

**Authors:** Callie E. Bounds, Steven A. Kwilas, Ana I. Kuehne, Jennifer M. Brannan, Russell R. Bakken, John M. Dye, Jay W. Hooper, Lesley C. Dupuy, Barry Ellefsen, Drew Hannaman, Hua Wu, Jin-an Jiao, Eddie J. Sullivan, Connie S. Schmaljohn

**Affiliations:** 1 United States Army Medical Research Institute of Infectious Diseases, Fort Detrick, Maryland, United States of America; 2 SAB Biotherapeutics, Sioux Falls, South Dakota, United States of America; 3 Ichor Medical Systems, Inc., San Diego, California, United States of America; Thomas Jefferson University, UNITED STATES

## Abstract

DNA vaccination of transchromosomal bovines (TcBs) with DNA vaccines expressing the codon-optimized (co) glycoprotein (GP) genes of Ebola virus (EBOV) and Sudan virus (SUDV) produce fully human polyclonal antibodies (pAbs) that recognize both viruses and demonstrate robust neutralizing activity. Each TcB was vaccinated by intramuscular electroporation (IM-EP) a total of four times and at each administration received 10 mg of the EBOV-GP_co_ DNA vaccine and 10 mg of the SUDV-GP_co_ DNA vaccine at two sites on the left and right sides, respectively. After two vaccinations, robust antibody responses (titers > 1000) were detected by ELISA against whole irradiated EBOV or SUDV and recombinant EBOV-GP or SUDV-GP (rGP) antigens, with higher titers observed for the rGP antigens. Strong, virus neutralizing antibody responses (titers >1000) were detected after three vaccinations when measured by vesicular stomatitis virus-based pseudovirion neutralization assay (PsVNA). Maximal neutralizing antibody responses were identified by traditional plaque reduction neutralization tests (PRNT) after four vaccinations. Neutralizing activity of human immunoglobulins (IgG) purified from TcB plasma collected after three vaccinations and injected intraperitoneally (IP) into mice at a 100 mg/kg dose was detected in the serum by PsVNA up to 14 days after administration. Passive transfer by IP injection of the purified IgG (100 mg/kg) to groups of BALB/c mice one day after IP challenge with mouse adapted (ma) EBOV resulted in 80% protection while all mice treated with non-specific pAbs succumbed. Similarly, interferon receptor 1 knockout (IFNAR ^-/-^) mice receiving the purified IgG (100 mg/kg) by IP injection one day after IP challenge with wild type SUDV resulted in 89% survival. These results are the first to demonstrate that filovirus GP DNA vaccines administered to TcBs by IM-EP can elicit neutralizing antibodies that provide post-exposure protection. Additionally, these data describe production of fully human IgG in a large animal system, a system which is capable of producing large quantities of a clinical grade therapeutic product.

## Introduction

Ebola virus (EBOV) and Sudan virus (SUDV) are non-segmented, negative strand RNA viruses belonging to the genus *Ebolavirus* of the family *Filoviridae*. Filoviruses cause acute hemorrhagic fever in humans and nonhuman primates (NHP) with symptoms of disease including sudden onset of fever, chills, headache, and anorexia followed by sore throat, vomiting, diarrhea, hemorrhaging, and the appearance of a petechial rash [1–4]. EBOV and SUDV are responsible for large and deadly outbreaks of hemorrhagic fever, with case fatality rates ranging from 30% to 90% [2]. The most recent and ongoing outbreak of EBOV in western Africa and the transport of infected people to other areas of the world have clearly established that filoviruses pose a significant disease threat that is not limited to the African continent. Additionally, there remains concern about the potential for deliberate misuse of these naturally occurring pathogens. Because filoviruses pose a high risk to public health and national security, the National Institute of Allergy and Infectious Diseases and Centers for Disease Control and Prevention have categorized them as Category A priority agents. In addition, the US Food and Drug Administration has not yet licensed prophylactic or therapeutic treatments for filovirus infections in humans. As a result, development of effective medical countermeasures remains a top priority.

One promising countermeasure is the use of antibodies to prevent or treat filovirus infections. Convalescent sera have been previously used to treat infected patients of sporadic outbreaks of hemorrhagic fever caused by filoviruses with mixed results [5, 6]. In laboratory studies, treatment with multiple doses of KZ52, a single human monoclonal antibody (mAb) derived from an EBOV survivor, prevented EBOV disease in guinea pigs [7]; however, follow up studies in NHP failed to show protection [8]. More recently, studies have demonstrated that a multi-dose regiment of polyclonal IgG purified from convalescent sera of vaccinated NHP that subsequently survived challenge, protect naïve NHP against filovirus challenge when given up to 48 hours post exposure [9]. Additionally, two groups showed that treatment of NHP with mouse/human chimeric mAbs up to 24 hours after filovirus challenge was partially protective [10, 11]. Another group reported complete protective immunity with the ZMab cocktail of murine mAbs in filovirus-infected NHP [12]. Likewise, a cocktail of three humanized antibodies, ZMapp, was 100% protective in NHP when administered up to 5 days after challenge [13]. Furthermore, this same cocktail was used to treat two American medical workers who contracted EBOV during the West African outbreak [14]. These studies have led to increased interest in systems that can be used to produce clinical-grade human antibodies for the prevention and treatment of filovirus infections. Genetically engineered transchromosomal bovines (TcBs) offer one possible means to achieve this goal.

TcBs genetically engineered to have the bovine heavy and lambda light chain loci knocked out and to express the entire non-rearranged human immunoglobulin heavy-chain and kappa light-chain loci from a human artificial chromosome [15–17] are capable of producing large amounts of fully human antigen-specific polyclonal antibodies (pAbs) [18]. As such, the generated IgG products overcome the significant limitation associated with IgG products obtained from other animal sources including toxicity upon repeated administrations as well as reduced antibody half- life due to clearance by the human host. Here we report the use of EBOV and SUDV DNA vaccines delivered by IM-EP to TcBs as a means of generating a fully human candidate polyclonal IgG product for these filoviruses. We also describe the results of studies to characterize the virus-binding and virus-neutralizing activity of these pAbs and to evaluate their ability to passively protect mice from otherwise lethal challenges with EBOV and SUDV.

## Results

### Production of human pAbs in TcBs vaccinated by IM-EP with DNA vaccines

**Fig 1:**
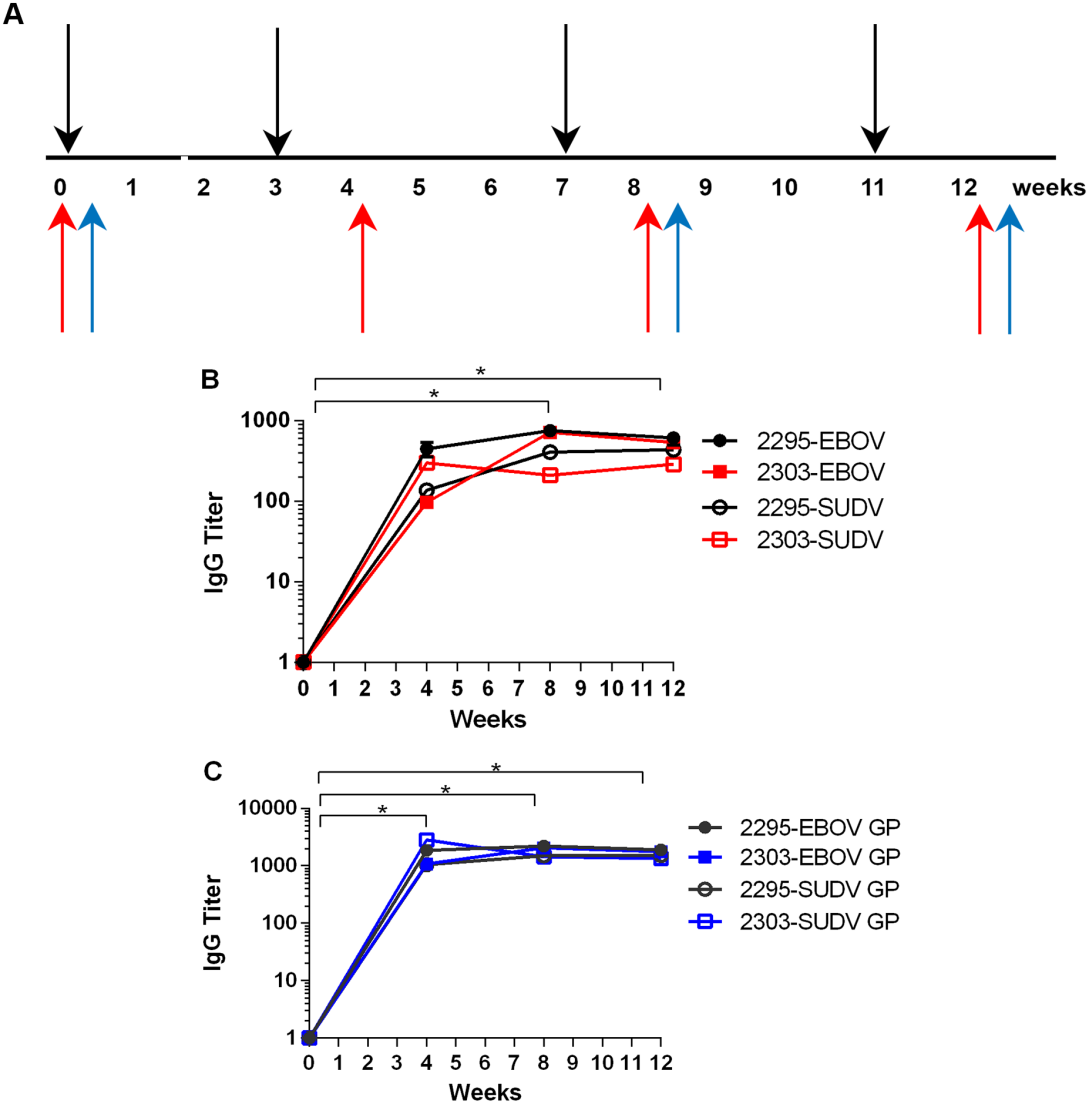
Production of human antibodies in TcBs. (A) Timeline of the vaccinations (black arrows), blood collections (red arrows) and plasma collections (blue arrows) for the TcBs. Serum samples obtained from two TcBs (#2295 and #2303) before vaccination (week 0) or 8–10 days after vaccinations 2–4 with EBOV-GP_co_ and SUDV-GP_co_ DNA vaccines were analyzed for total IgG antibodies by ELISA using (B) whole irradiated EBOV or SUDV antigens, and (C) EBOV rGP or SUDV rGP. Symbols represent the titers at each time point for each TcB.

Two TcBs (#2295, #2303) were each vaccinated four times at 3- to 4-week intervals with 10 mg each of the DNA vaccines expressing codon-optimized glycoprotein (GP) genes (EBOV-GP_co_, SUDV-GP_co_) delivered by IM-EP (Fig 1A). Serum samples collected from each TcB prior to the first vaccination (week 0) served as negative controls for *in vitro* assays. Additionally, antibodies purified from large volumes of plasma collected from each TcB before the first and after the third vaccinations were used for the passive transfer studies as non-specific (NS) pAbs and EBOV/SUDV pAbs, respectively.

One week following the second vaccination (week 4), serum samples collected from each TcB displayed antibodies against EBOV and SUDV as assessed by ELISA using irradiated whole virus or recombinant EBOV-GP or SUDV-GP as antigens (Fig 1B and 1C). Antibody responses produced by both TcBs against EBOV- and SUDV-rGP were significantly (p < 0.0001) higher after the second vaccination (week 4) when compared with the pre-vaccination sera controls and these titers remained significantly high (week 8 p < 0.0001 and week 12 p < 0.0001) in both TcBs through the fourth vaccination. Additionally, antibody responses generated in both TcBs against irradiated whole EBOV and SUDV viruses were significantly (p <0.01) higher after the third vaccination (week 8) when compared with pre-vaccination sera controls, and these titers remained significantly (p < 0.001) high in both TcBs through the fourth vaccination (week 12). Maximal antibody responses generated in each TcB to the recombinant GPs were reached after the second (TcB # 2303) or third (TcB #2295) vaccination but remained within the dynamic range of the ELISA as positive control human sera or the human mAb KZ52 displayed higher end point titers (data not shown).

### Neutralizing antibody responses generated in DNA-vaccinated TcBs

To assess the virus-neutralizing antibody responses generated in the vaccinated TcBs, we performed pseudovirion neutralization assays (PsVNA) using vesicular stomatitis virus (VSV) pseudotyped with the GP proteins of EBOV or SUDV [19–21] as well as traditional plaque reduction neutralization tests (PRNT) [22]. Both assays provide relevant information in that the pseudovirion assay is the method used for assessing human responses in ongoing vaccine studies [23], while PRNT has been used in numerous past animal studies. Both TcBs generated serum neutralizing antibody responses to EBOV and SUDV (Table 1) after the second vaccination (week 4), which is congruent with the total IgG anti-GP titers. Robust neutralizing antibody responses (titers >1000), which were significantly higher than pre-vaccination control sera were detected by PsVNA in both TcBs against both EBOV (2295 *p* = 0.004; 2303 *p* = 0.0295) and SUDV (2295 *p* < 0.001; 2303 *p* = 0.0009) after the third vaccination (week 8) and these responses remained significantly higher than controls after the fourth vaccination (week 12) against EBOV (2295 *p* < 0.0001; 2303 *p* = 0.003) and SUDV (2295 *p* = 0.0001; 2303 *p* < 0.0001). Serum samples were evaluated in parallel by PRNT to confirm the results observed by PsVNA. The PRNT and PsVNA results depict similar trends with neutralizing antibody responses increasing after each vaccination, although neutralizing antibody responses detected by PRNT were much lower.

**Table 1:** Serum neutralizing antibody responses of TcBs after vaccinations 2–4.

PsVNA_80_ Titers (EBOV/SUDV)				PRNT_80_ Titers (EBOV/SUDV)		
Animal ID #	Week 4	Week 8	Week 12	Week 4	Week 8	Week 12
# 2295 serum	984/522	3287/2696	4147/2259	10/10	20/20	160/40
# 2303 serum	440/1173	1884/1828	3296/4042	ND^a^/20	10/20	160/80

^a^ND: not detected

### Characterization of purified EBOV/SUDV human pAbs

**Fig 2:**
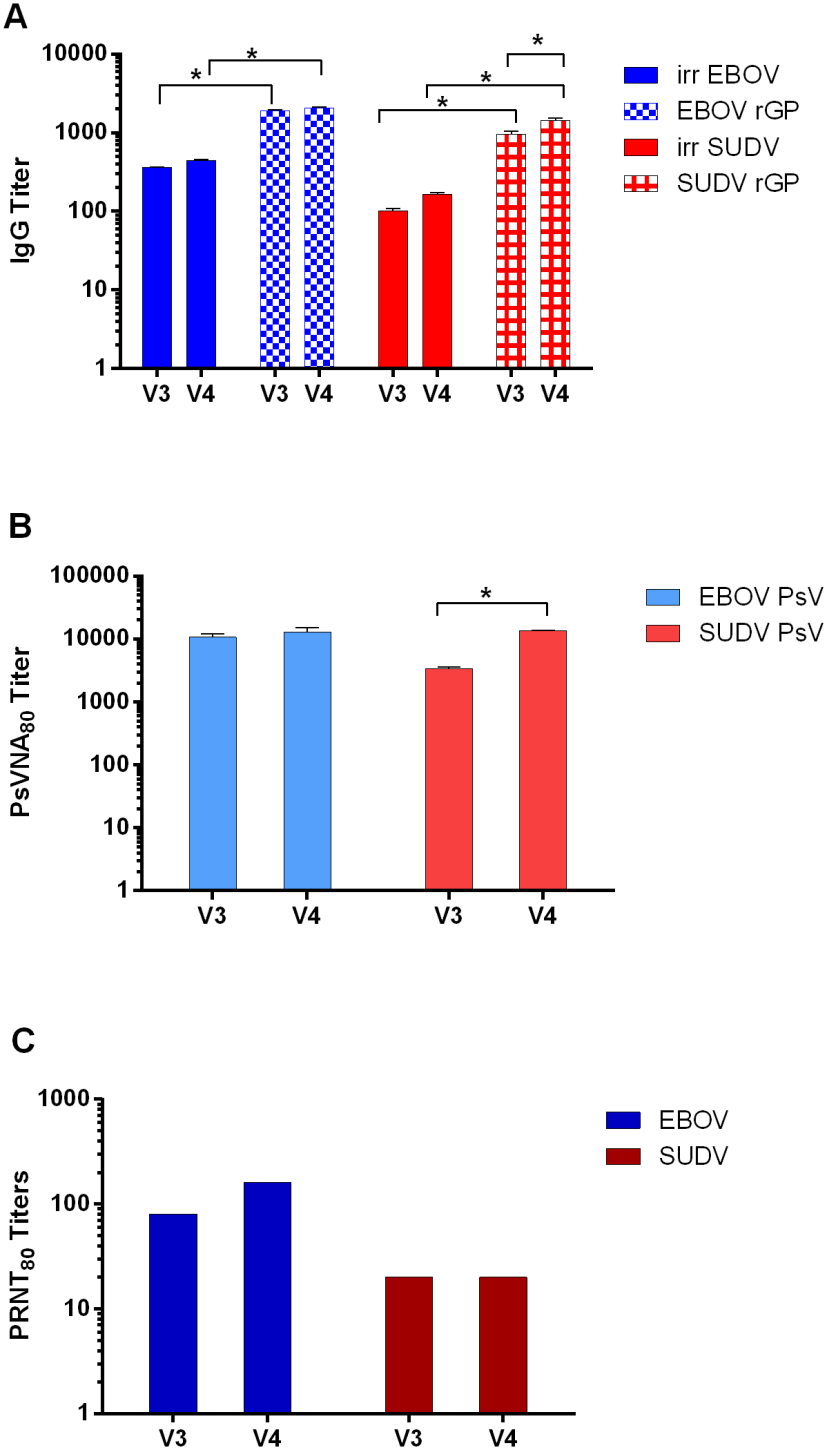
Purified pAbs from vaccinated TcBs demonstrate neutralizing activity against EBOV and SUDV. Antibodies were purified from plasma collected from both TcBs eight days following the third and fourth vaccinations. (A) These purified V3 (22.16 mg/ml) and V4 (36.68 mg/ml) EBOV/SUDV human pAbs were evaluated by standard ELISA for total IgG antibody responses using whole-irradiated EBOV or SUDV antigen, and EBOV rGP or SUDV rGP antigen. (B) EBOV- and SUDV- neutralizing activity of the purified EBOV/SUDV human pAbs was determined by PsVNA, (C) and PRNT. Significant differences between titers are denoted by (*) where *p* < 0.05.

Fully human IgG was purified from large quantities of plasma collected from each vaccinated TcB eight days after the third (V3) and fourth (V4) vaccinations. The total protein concentrations of the final purified IgG preparations for the V3 and V4 materials were 22.16 mg/ml and 36.68 mg/ml, respectively. To verify the virus-binding capacity of the purified pAbs, total IgG anti-EBOV and anti-SUDV antibodies were measured by ELISA using whole-irradiated virus or rGP antigens. Consistent with the results obtained with the serum samples collected from these vaccinated TcBs, total IgG responses of the V3 and V4 EBOV/SUDV human pAb samples were significantly higher against the EBOV rGP (p < 0.0001) and SUDV rGP (p < 0.0001) antigens as compared to the whole-irradiated EBOV and SUDV antigens (Fig 2A). Furthermore, the V4 EBOV/SUDV human pAbs had titers significantly (*p* = 0.0084 and *p* < 0.0001) higher when EBOV rGP or SUDV rGP were used as coating antigens compared to the V3 EBOV/SUDV human pAbs.

To confirm the virus-neutralizing activity of the purified V3 and V4 EBOV/SUDV human pAbs, neutralizing titers against EBOV and SUDV were determined by PsVNA and PRNT. The PsVNA_80_ titers of the purified V3 EBOV/SUDV human pAbs against both EBOV and SUDV were 10520 and 3260, respectively (Fig 2B). The PsVNA_80_ neutralizing titers of the purified V4 EBOV/SUDV human pAbs remained relatively the same against EBOV at 12866, while the PsVNA_80_ titer against SUDV significantly (*p* = 0.0032) increased to 14583. The PRNT_80_ titers of the purified V3 EBOV/SUDV human pAbs were 80 and 20 against EBOV and SUDV, respectively (Fig 2C). The PRNT_80_ titers of the purified V4 EBOV/SUDV human pAbs increased to 160 against EBOV while the SUDV titer remained 20.

### Bioavailability of passively transferred purified human EBOV/SUDV pAbs in mice

**Fig 3:**
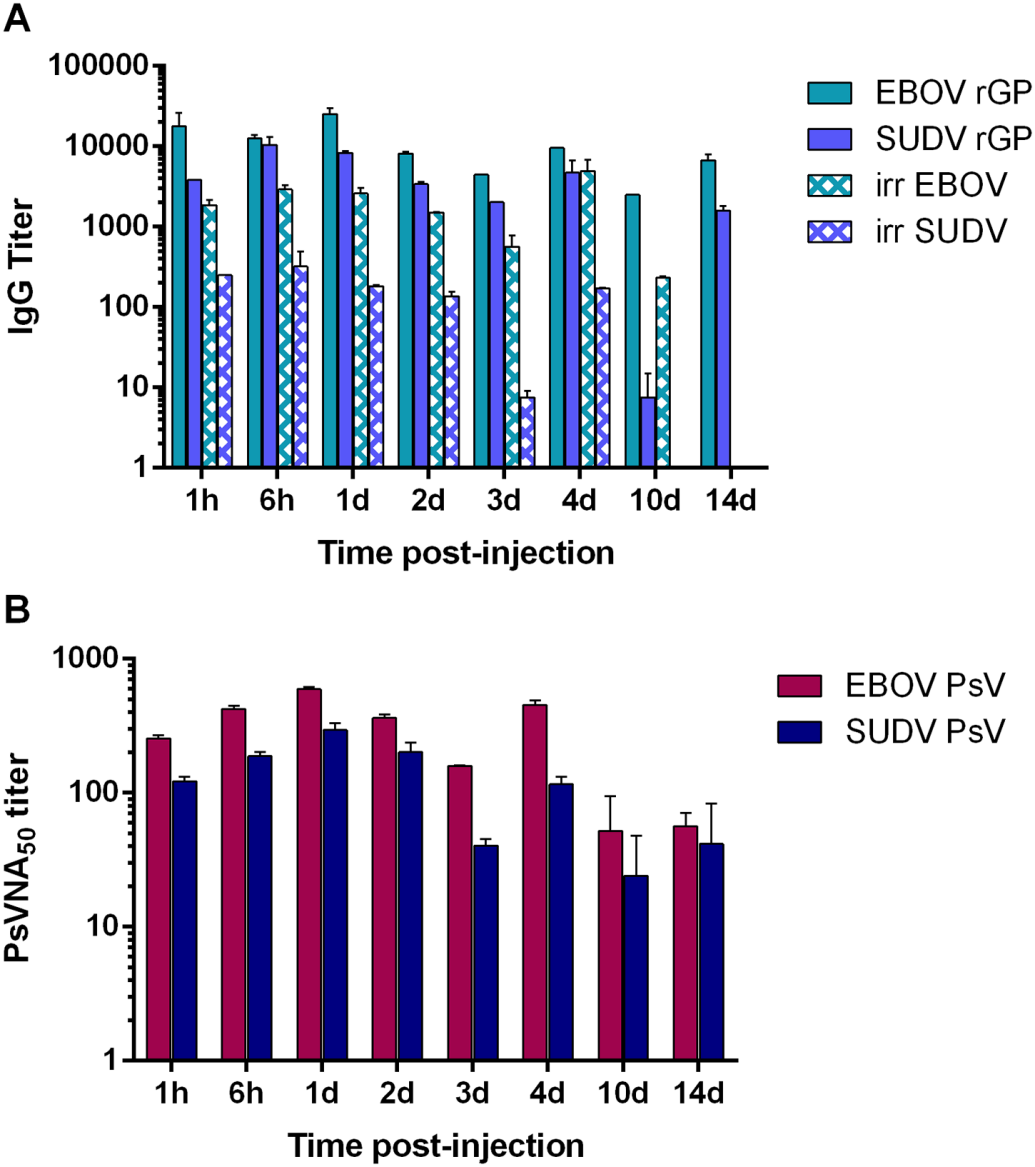
Bioavailability of purified EBOV/SUDV human pAbs in mice. BALB/c mice (N = 10) received a single IP injection of 100 mg/kg of the purified V3 EBOV/SUDV human pAbs. (A) Sera collected from individual mice at the indicated time points after injection were analyzed by standard ELISA for total human IgG using whole-irradiated EBOV or SUDV antigen, and EBOV rGP or SUDV rGP antigen. (B) Serum samples were also evaluated for EBOV- and SUDV-neutralizing activity by PsVNA.

To assess the bioavailability of the purified EBOV/SUDV human pAbs in mice, we passively transferred 100 mg/kg of the purified V3 EBOV/SUDV human pAbs into mice by IP injection. Serum samples were obtained 1h and 6h after injection and on days 1, 2, 3, 4, 7, 10, and 14 after administration of the pAbs. The persistence of the anti-EBOV and anti-SUDV antibodies was monitored by ELISA using irradiated whole virus or recombinant EBOV-GP or SUDV-GP as antigens while neutralizing activity was monitored by PsVNA (Fig 3A and 3B). The purified V3 EBOV/SUDV human pAbs were detected by ELISA out to days 4 and 10 when irradiated whole SUDV or EBOV was used as the antigen, respectively. The human pabs could be detected by ELISA out to day 14 when recombinant EBOV-GP or SUDV-GP was used as the antigen. Neutralizing activity against both EBOV and SUDV peaked 24 hours after administration but could still be detected out to day 14.

### Protective efficacy of passively transferred purified EBOV/SUDV human pAbs in mice

**Fig 4:**
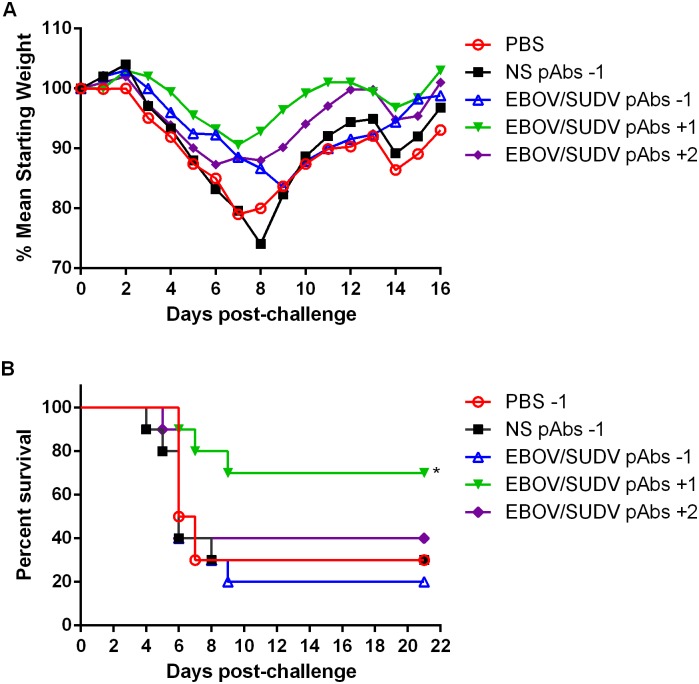
Weight loss and survival of BALB/c mice challenged with maEBOV when administered purified EBOV/SUDV human pAbs before or after challenge. Groups of BALB/c mice (N = 10) received a single IP injection of PBS, 100mg/kg NS pAbs, or 100mg/kg EBOV/SUDV pAbs one day before, or one or two days after challenge with 1000 pfu of maEBOV by IP injection. (A) Mean weight was determined daily for each dosing group and graphed as the percent mean of the starting weight. (B) Kaplan-Meier survival curves indicating the percentage of surviving mice at each day of the 21-day post-challenge observation period are shown. Survival of mice receiving the NS pAbs compared to the EBOV/SUDV pAbs one day before challenge (*p* = 0.9573), NS pAbs vs EBOV/SUDV pAbs one day after challenge (*p* = 0.0449), and NS pAbs vs EBOV/SUDV pAbs two days after challenge (*p* = 0.5720). Significant differences between survival curves are denoted by (*) where *p* < 0.05.

To assess the protective efficacy of the purified EBOV/SUDV human pAbs produced in the vaccinated TcBs against EBOV infection, groups of 6–8 week old BALB/c mice (N = 10) were challenged by IP injection with 1000 plaque forming units (pfu) of mouse adapted (ma) EBOV as previously described [24]. Control groups of mice received phosphate buffered saline (PBS) or 100 mg/kg of NS pAbs by IP injection one day prior to challenge. Three experimental groups of mice received single 100 mg/kg doses of the purified V3 EBOV/SUDV human pAbs by IP injection one day before, one day after or two days after challenge. Mice were observed for clinical signs of EBOV infection and all mice across all groups displayed reduced grooming and subdued behavior; these clinical signs were accompanied by an increase in weight loss (Fig 4A). Seven of the 10 mice treated with the V3 EBOV/SUDV human pAbs one day after challenge survived (Fig 4B). There was no significant difference in the survival of control mice (30% survival in both groups) or mice receiving the V3 EBOV/SUDV antibodies one day before (20% survival) or two days after (40% survival) challenge.

We performed a second passive protection study in mice to confirm the protective efficacy of the purified V3 EBOV/SUDV human pAbs one day after challenge and to assess the effects of reduced and serial dosing. Because of the incomplete lethality in the control groups of mice in our first study, we also used a lower challenge dose of 100 pfu of maEBOV, which has been found by others to be more uniformly lethal in BALB/c mice, possibly due to the presence of fewer defective interfering (DI) viruses in the stock [25]. One day after challenge, groups of BALB/c mice (N = 10) received a single dose of the V3 EBOV/SUDV pAbs at 100 mg/kg, 50 mg/kg, or 10 mg/kg. A control group received a single dose at 100 mg/kg of the NS pAbs. To examine the effects of sequential dosing, we included groups that received 50 mg/kg, 25 mg/kg, or 5 mg/kg of the V3 EBOV/SUDV pAbs on days 1 and 2 following challenge.

**Fig 5:**
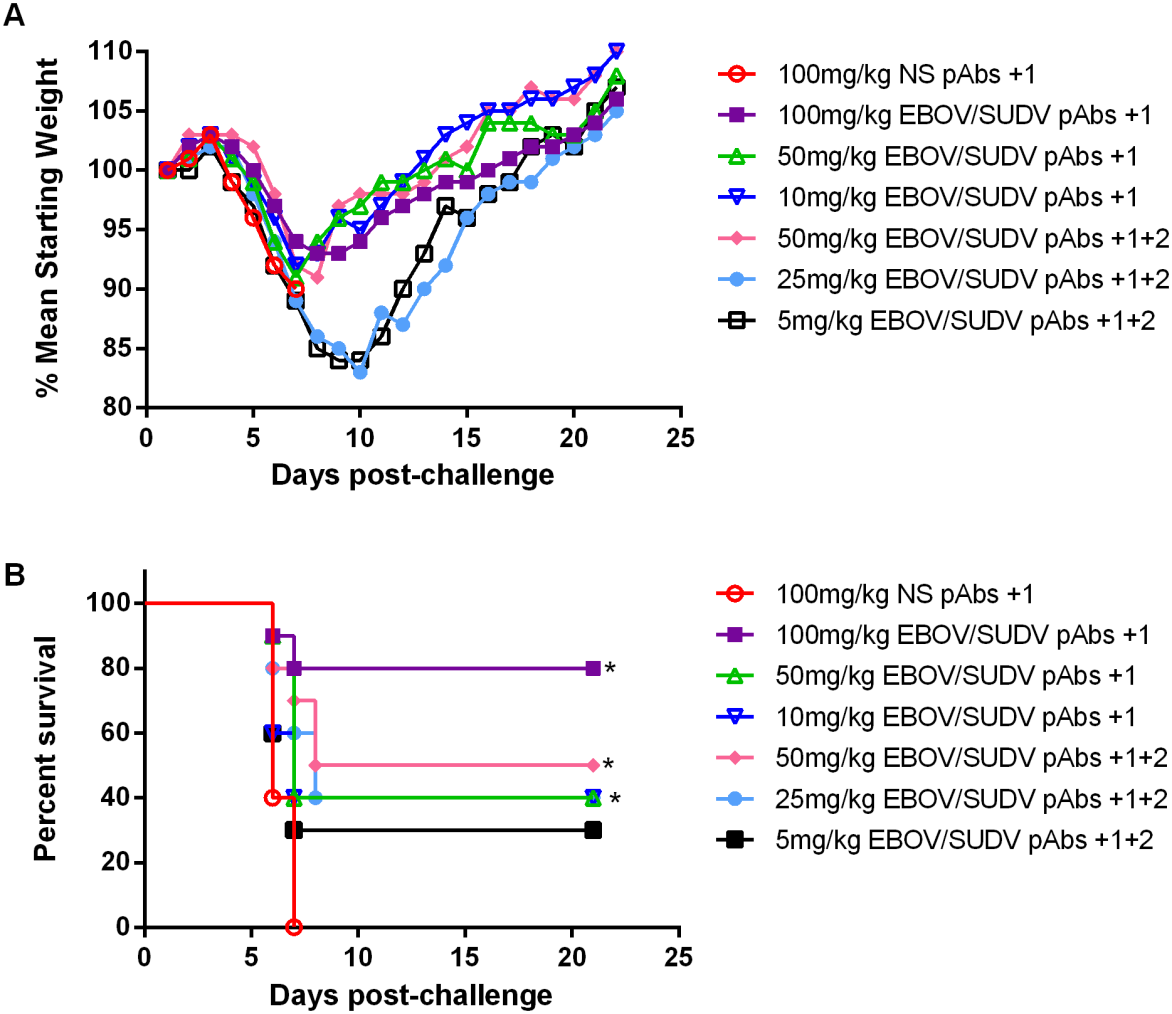
Weight loss and survival of BALB/c mice administered high, medium, or low doses of purified EBOV/SUDV human pAbs after maEBOV challenge. Groups of BALB/c mice (N = 10) received a single IP injection of 100mg/kg NS pAbs, 100 mg/kg EBOV/SUDV pAbs, 50 mg/kg EBOV/SUDV pAbs, or 10 mg/kg EBOV/SUDV pAbs one day after challenge or 50 mg/kg EBOV/SUDV pAbs, 25 mg/kg EBOV/SUDV pAbs, or 5 mg/kg EBOV/SUDV pAbs on day 1 and day 2 after challenge with 100 pfu maEBOV. (A) Mean weight was determined daily for each dosing group and graphed as the percent mean of the starting weight. All mice receiving the NS pabs succumb by day 8 post-challenge. (B) Kaplan-Meier survival curves indicating the percentage of surviving mice at each day of the 21-day post-challenge observation period are shown. The p-values for the following comparisons are as follows: 100 mg/kg NS pAbs vs. 100 mg/kg EBOV/SUDV pAbs +1 (*p* = 0.0003), 100 mg/kg NS pAbs vs. 50 mg/kg EBOV/SUDV pAbs +1 (*p* = 0.0059), NS pAbs vs. 10 mg/kg EBOV/SUDV pAbs +1 (*p* = 0.0630), NS pAbs vs. 50 mg/kg EBOV/SUDV pAbs +1 and +2 (*p* = 0.002), NS pAbs vs. 25 mg/kg EBOV/SUDV pAbs +1 and +2 (*p* = 0.0046), NS pAbs vs. 5mg/kg EBOV/SUDV pAbs +1 and +2 (*p* = 0.1082), 100mg/kg EBOV/SUDV pAbs +1 vs. 50 mg/kg EBOV/SUDV pAbs +1 and +2 (*p* = 0.1988), 50 mg/kg EBOV/SUDV +1 vs. 25 mg/kg EBOV/SUDV pAbs +1 and +2 (*p* = 0.9968), and 10 mg/kg EBOV/SUDV pAbs +1 vs. 5 mg/kg EBOV/SUDV pAbs +1 and +2 (*p* = 0.7275). Significant differences between survival curves are denoted by (*) where *p* < 0.05.

Clinical signs of EBOV infection, including weight loss, were observed beginning on days 4 or 5 in all groups of mice and by day 8 all mice receiving a 100 mg/kg dose of the NS pabs succumbed to disease (Fig 5A). Eight of the 10 mice that received a single 100 mg/kg dose of the V3 EBOV/SUDV pAbs one day after challenge survived (Fig 5B). Mice that received 50 mg/kg dose of the V3 EBOV/SUDV human pAbs on days 1 and 2 following challenge had a survival rate of 50%, which was significantly higher than controls (*p* = 0.0059), and was not significantly less than those receiving the 100 mg/kg dose (*p* = 0.199). Additionally, mice that received a single 50 mg/kg dose of the EBOV/SUDV pAbs one day post challenge or serial doses of 25 mg/kg on days one and two post challenge had a survival rate of 40%, which was also still significantly higher (*p* = 0.0059; *p* = 0.0046) than those receiving the NS pAbs. Mice receiving a single 10 mg/kg dose of the V3 EBOV/SUDV human pAbs one day post challenge and mice receiving serial doses at 5 mg/kg on days one and two after challenge did not have survival rates that were statistically significant when compared to mice receiving the NS pAbs.

**Fig 6:**
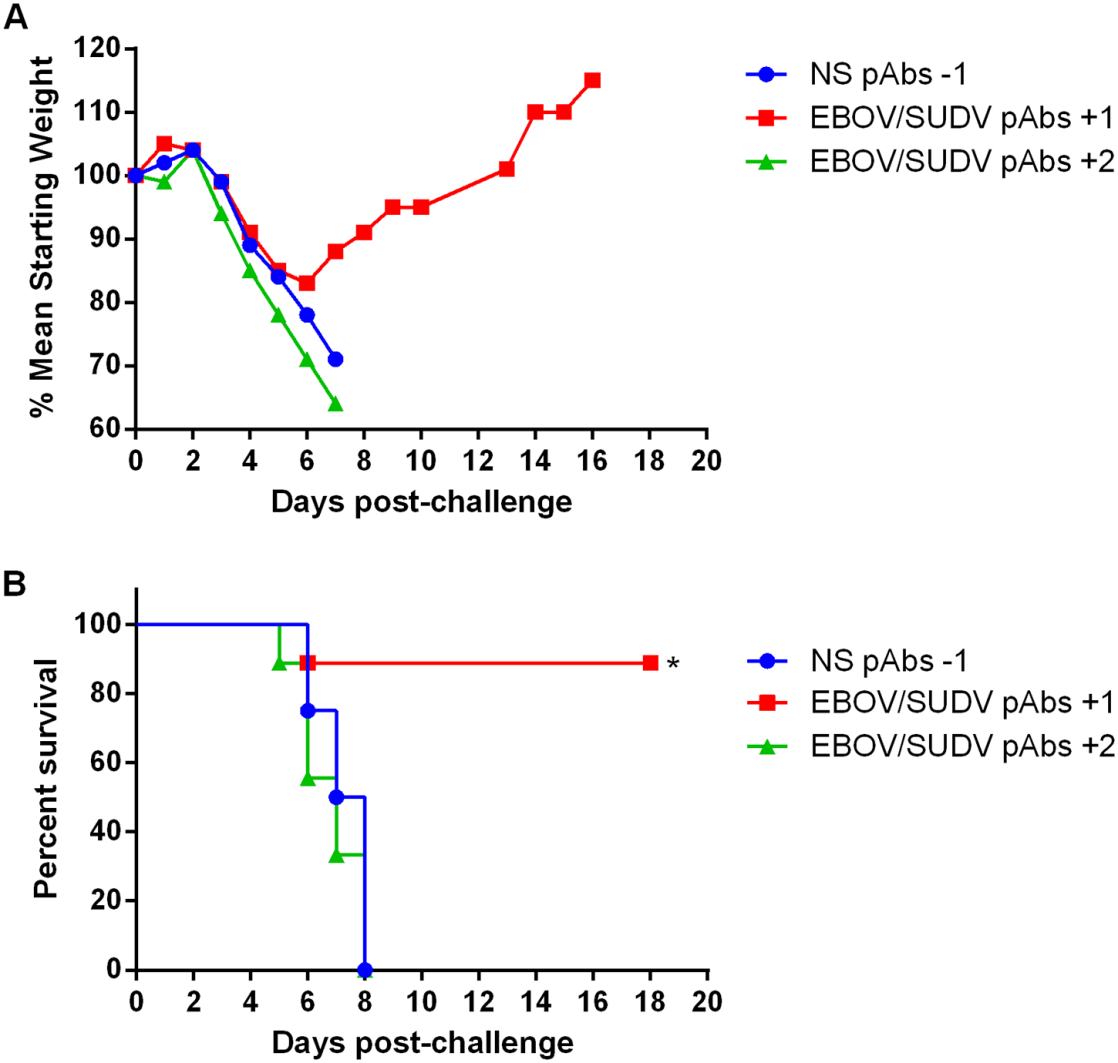
Weight loss and survival of IFNR ^-/-^ mice challenged with SUDV when administered purified EBOV/SUDV human pAbs after challenge. Groups of IFNR -/- mice (N = 9) received a single IP injection of 100 mg/kg NS pAbs one day before, or 100 mg/kg EBOV/SUDV pAbs one day or two days after challenge via the IP route with 1000 pfu of SUDV. (A) Mean weight was determined daily for each dosing group and graphed as the percent mean of the starting weight. All mice in the NS pabs or the EBOV/SUDV pabs +2 groups succumb to disease by day 8 post-challenge. (B) Kaplan-Meier survival curves indicating the percentage of surviving mice at each day of the 19-day post-challenge observation period are shown. Survival of mice receiving the NS pAbs compared to the EBOV/SUDV pAbs one day after challenge (*p* = 0.0009), and NS pAbs versus EBOV/SUDV pAbs two days after challenge (*p* = 0.3981). Significant differences between survival curves are denoted by (*) where *p* < 0.05.

Although a mouse adapted strain of SUDV has not been reported, wild type SUDV has been shown to be lethal in interferon receptor knockout (IFNAR ^-/-^) mice [26–28]. To evaluate the protective efficacy of the purified V3 EBOV/SUDV human pAbs against SUDV challenge, groups of 4-week old IFNAR ^-/-^ mice (N = 9) were challenged by IP injection with 1000 pfu of wild type SUDV. Groups received single IP injections of a 100 mg/kg dose of the V3 EBOV/SUDV human pAbs one day after or two days after challenge. A negative control group received the NS pAbs at a dose of 100 mg/kg one day before challenge. Similar to maEBOV challenge, all mice displayed clinical signs of SUDV infection, including weight loss, starting on days 4 or 5 (Fig 6A). Eight of 9 IFNAR *-/-* mice treated with the purified V3 EBOV/SUDV human pAbs starting one day after challenge survived (Fig 6B). In contrast, all mice that received the purified V3 EBOV/SUDV human pAbs two days after challenge or the NS pAbs one day before challenge succumbed.

## Discussion

We previously showed that when delivered by IM-EP, DNA vaccines expressing the codon-optimized GP genes of EBOV, SUDV, and Marburg virus (MARV) induce high levels of IgG antibodies and completely protect mice from EBOV and MARV challenge [29]. Additionally, robust antibody responses, including virus-neutralizing antibodies, were generated in cynomolgus macaques receiving these same DNA vaccines via IM-EP, and most macaques were protected against challenge with EBOV or MARV [21]. In addition to these studies, others have shown that IM-EP is an effective mode of DNA immunization in multiple animal species [30–32]. Also, DNA vaccination of TcBs by jet injection with a hantavirus DNA vaccine was able to elicit human anti-hantavirus polyclonal IgG capable of preventing lethal disease in Syrian hamsters infected with homologous hantavirus [18]. In the study presented here, we used the codon-optimized EBOV and SUDV GP DNA vaccines to immunize two TcBs by IM-EP. Our intent was to produce large quantities of a fully human polyclonal IgG product that would be suitable for immune therapy of filovirus infected humans. Within 10 weeks of initial vaccination of two TcBs, we were able to produce enough IgG product for treatment of approximately 80 NHP, based on a 3 dose regimen. Moreover, an improved TcB genotype has been developed which has been found to produce 2–3 times the amount of purified antibody as can be recovered from the TcB genotype used for our studies (SAB unpublished information). For these studies, both of the TcBs developed high titer antibody responses detected by ELISA and neutralizing assays. Purification of these human pAbs provided an IgG product that conferred significant post-exposure protection against lethal EBOV and SUDV challenges in mice. To our knowledge, this represents the first report of the successful use of this approach to generate a fully human filovirus-specific polyclonal antibody product.

Presently, we do not have a clear understanding of the mechanism by which the purified IgG protects against EBOV and SUDV. It is likely that neutralizing antibodies play a role in this protection; however, it is also possible that antibodies that do not neutralize virus in cell culture play a role in protection (e.g., antibody-dependent cell-mediated cytotoxicity, antibody-dependent complement-mediated virolysis). To begin to investigate the mechanism of protection we measured neutralizing antibody activity using two different assays, the PsVNA and PRNT. Our results from both assays show a similar trend with virus neutralization titers increasing after each vaccination, although the PRNT titers are considerably lower than the PsVNA titers. The reasons for this have not been fully explored, but there are several possibilities to explain these differences. It is possible that the larger dynamic range of the PsVNA provides increased sensitivity and allows detection of inhibition by GP-mediated entry in the presence of lower antibody levels. Another possibility is that less GP-specific antibody is required to neutralize the VSV pseudovirion than EBOV virions due to their size differences. The number of GP trimers on the surface of either type of particle is unknown, but it is likely that the larger EBOV particles (~80 x 800 nm) have more GP displayed on their surface than the smaller VSV particles (~ 70 x 200 nm), and would require a larger quantity of antibody to achieve neutralization. One more possibility is that neutralization of live EBOV could require higher quantities of neutralizing antibodies because non-neutralized virus can replicate and spread cell to cell, while pseudovirions are non-replicating particles. Also, there are fundamental differences between how the two assays are set up that could explain the differences. For example, pseudovirions are incubated at 4°C for 18–24 hours with test samples while EBOV is incubated at 37°C for 1 hour. It is possible that with the shorter incubation time of the PRNT weaker neutralizing responses would not have a large enough effect on virus entry to be detected. Conversely, the longer incubation time could result in weaker neutralizing responses having more of an effect on pseudovirion entry. These and other possibilities will likely become more clearly defined as attempts are made to validate the EBOV PsVNA, which is currently being used to assess the neutralizing antibody responses of vaccines in clinical trials [23].

In our initial EBOV passive transfer and challenge study, we observed reduced mortality in mice treated one day after challenge with 1000 pfu of maEBOV. However, in this study we observed incomplete lethality of the negative controls. We, and others, have seen this phenomenon in previous vaccine studies [29, 33] and this finding might be related to the presence of defective interfering (DI) particles in the high challenge dose. DI particles are subgenomic truncation mutants that require the parental virus to act as a helper for propagation; replication usually occurs at the expense of the infectious parental virus [34]. DI particles occur among many RNA viruses and can modulate the disease course through attenuation of the competent virus resulting in loss of virulence [35–37]. Additionally, DI particles have been shown to protect against lethal disease through modulation of the immune response [38, 39] and previous studies have demonstrated that EBOV DI particles are generated in cell culture [25]. Therefore, in the follow up maEBOV challenge study, we used a lower challenge dose which was completely lethal to controls. We confirmed the initial finding that it is possible to prevent death of mice by treating them with the pAbs derived from the vaccinated TcBs one day after challenge with maEBOV. We also showed that serial dosing spread over two days following infection was not as effective as giving a single large bolus of the pAbs.

Similar to our results with maEBOV, we found that passive transfer of the purified EBOV/SUDV human pAbs to mice one day after infection with SUDV protected them from disease and death. However, transfer two days after challenge was ineffective. Studies by others have shown increased survival of rodents challenged with maEBOV or guinea pig-adapted EBOV when treatment with mouse mAbs was initiated at 1 or 2 days post infection rather than 1 day before infection [40]. Taken together, these data indicate that in mice there is only a very limited treatment window available for initiation of therapy after filovirus infection. Earlier studies have shown individual mAbs and mAb cocktails can protect mice and guinea pigs from lethal challenge with filoviruses [7, 41]; however, neither of these animal models are always predictive of protection of NHP [8]. Clearly, it will be necessary to perform further studies to measure the efficacy of the human pAbs against EBOV and SUDV in NHP as well as measure the potential treatment windows. Additionally, the effects of treatment on viremia and virus dissemination will need to be investigated.

In summary, the data presented here provide a proof of concept that filovirus DNA vaccines can be used in concert with the TcB platform to produce a safe, biologically active, fully human polyclonal IgG product that offers post-exposure protection against filovirus infection.

## Materials and Methods

### DNA Vaccines

DNA vaccines expressing codon-optimized GP genes of EBOV and SUDV (EBOV-GP_co_, SUDV-GP_co_) were generated as previously described [29]. Briefly, these genes were synthesized by GeneArt and cloned into the *NotI* and *BglII* restriction sites of the pWRG7077 eukaryotic expression vector. Research grade plasmids were manufactured by Aldevron (Fargo, ND).

### Transchromosomal bovines (TcBs)

The TcBs used in this study are triple knockouts for the endogenous bovine immunoglobulin genes, (bIGHM^-/-^, bIGHML1^-/-^, bIGL^-/-^), and contain a human artificial chromosome (HAC) vector labeled KcHACD [15–17]. This HAC vector expresses fragments of human chromosome (HC) 14 and HC 2. The HC 14 fragment contains the entire human immunoglobulin heavy chain locus with the exception of the immunoglobulin heavy constant mu (IGHM) region which remains bovine. The HC 2 fragment contains the human immunoglobulin kappa light-chain (Igk) locus.

### TcB vaccinations

Two TcBs were vaccinated with both the EBOV-GP_co_ DNA vaccine and the SUDV-GP_co_ DNA vaccine by IM-EP using the TriGrid Delivery System (TDS; Ichor Medical Systems) at 3–4 week intervals as previously described [42]. Briefly, TcBs were anesthetized with a mixture of xylazine and ketamine, the TriGrid electrode array with 8-mm spacing containing a 22 gauge syringe loaded with a 1ml DNA solution inserted through the central injection port was inserted either on the neck or on the hind leg, and the automatic injection device was activated. Following DNA injection a 250v/cm electrical field was applied locally for a total duration of 40-ms over a 400-ms interval. The EBOV-GP_co_ DNA vaccine was administered on the left side of each animal while the SUDV-GP_co_ DNA vaccine was administered on the right side of each animal. Both vaccines were administered at 5mg/ml for a total dose of 10mg/vaccine/TcB vaccination. To enhance the immune responses, SAB’s proprietary adjuvant formulation (SAB-adj-1) was injected at sites adjacent to the DNA vaccination sites by standard IM injection. The adjuvant formulation was administered in a 1-ml volume.

### Purification of fully human IgG

Plasma collected from both TcBs prior to the first vaccination was the source material for the fully human negative control pAbs. Plasma collected from each vaccinated TcB on day 8 following the third vaccination was the source material for the fully human EBOV/ SUDV pAbs used in the challenge studies though human EBOV/SUDV pabs purified from plasma collected from each vaccinated TcB on day 8 following the fourth vaccination was also analyzed by *in vitro* assays. Purified IgG was obtained from the plasma as previously described [18, 43]. Briefly, frozen plasma was thawed overnight at 25°C, pooled, and the pH adjusted to 4.8 with 20% acetic acid. The plasma was then fractionated at low pH with caprylic acid followed by a filtration step using a depth filter device to remove non-IgG proteins from bovine plasma. The filtrate was then adjusted to a pH of 7.5 with 1M Tris and further purified by using human IgG light chain kappa specific affinity chromatography followed by a second purification using bovine IgG heavy chain specific affinity chromatography. The purified EBOV/SUDV IgG has a protein concentration of 22.16 mg/ml (from the third vaccination) or 36.68 mg/ml (from the fourth vaccination) in a sterile liquid containing 10 mM glutamic acid monosodium salt, 262 mM D-sorbitol, 0.05 mg/mL Tween80, pH 5.5.

### ELISA

Total IgG anti-EBOV and anti-SUDV endpoint antibody titers were determined for serum samples and the purified pAbs by standard enzyme-linked immunosorbent assay (ELISA) using sucrose gradient-purified, EBOV or SUDV virions which were inactivated by gamma irradiation, or commercially available recombinant EBOV or SUDV GP (IBT Bioservices) as previously described by [9, 44]. Briefly, recombinant GP antigens were diluted in PBS (2 μg/ml) or whole irradiated EBOV or SUDV antigens were diluted in PBS (1 μg/ml and 0.44 μg/ml) and 50 μl/well was added to polystyrene plates (Costar 3590). Plates were incubated at 4°C overnight. Antigen was removed prior to blocking at room temperature for 2 hours with a solution of 5% milk in PBS/0.05% Tween 20. TcB serum samples or purified pAbs were diluted in blocking buffer supplemented with 1% goat serum (42.8 μg/ml) and serial 0.5-log dilutions were performed. Antigen-coated ELISA plates were incubated with diluted samples for 2 hours at room temperature and then washed with wash buffer (PBS, 0.05% Tween 20). A gamma chain-specific mouse anti-human horseradish peroxidase (HRP)-conjugated secondary antibody (mybiosource.com) was diluted in blocking buffer, added to the plates and incubated for 1 hour at room temperature. Plates were washed and an ABTS peroxidase substrate (KPL) was added for 25–30 minutes at room temperature followed by ABTS Stop solution (KPL). The optical density at 405nm was measured for all plates using a SpectraMax M2e microplate reader (Molecular Devices). End point titers were calculated using Softmax Pro v5.4.1 (Molecular Devices).

### Pseudovirion Neutralization Assay (PsVNA)

Pseudovirions (PsV) that express luciferase were prepared in HEK 293T cells as previously described by Kwilas et. al. [19] using the EBOV-GP_co_ and SUDV-GP_co_ DNA vaccine plasmids to express the filovirus GPs. Serum samples collected from the vaccinated TcBs were heat inactivated at 56°C for 30 min and then an initial 1:10 dilution of the heat inactivated sera or purified material was made followed by five-fold serial dilutions. Samples were diluted in complete Eagle’s minimum essential medium with Earle’s salts (cEMEM) containing 10% heat inactivated FBS, 10mM Hepes (pH 7.4), 100 IU/ml penicillin, and 100 μg/ml streptomycin and analyzed in triplicate. An equal volume of cEMEM supplemented with 10% human complement (Sigma) containing 4000 focus-forming units of each filovirus PsV was added to the sera dilutions and incubated overnight at 4°C. Following incubation, Vero cell monolayers seeded in flat, clear bottom black-walled 96-well plates (Corning) were inoculated with 50μl of the PsV:TcB serum mixture and incubated at 37°C for 18–24 hours. The medium was discarded, the cells were lysed, and luciferase substrate was added according to the *Renilla* Luciferase Assay System protocol (Promega #E2820). The luciferase data were acquired using a Tecan M200 Pro microplate reader. The raw data were graphed using GraphPad Prism (version 6) to calculate percent neutralization. The data were fit to a four-parameter logistic curve and the PsVNA_80_ neutralization titers were interpolated.

### Plaque Reduction Neutralization Test (PRNT)

Serum samples collected from the vaccinated TcBs were heat inactivated at 56°C for 30 min and serum or purified samples were diluted to a working concentration of 2 mg/ml. Initial 1:5 dilutions of the samples were made followed by two-fold serial dilutions. Samples were diluted in complete Eagle’s minimum essential medium with Earle’s salts containing 2% heat inactivated FBS and 0.05% Gentamicin and analyzed in duplicate. An equal volume of cEMEM supplemented with 10% guinea pig complement (Cedarlane) containing 100 pfu of EBOV or SUDV was added to the sera dilutions and incubated at 37°C for 1 hour. Following incubation, Vero or Vero E6 cell monolayers were inoculated, overlaid with agarose and incubated at 37°C. A second agarose overlay containing 5% neutral red was added 7 days (EBOV) or 8 days (SUDV) later and plaques were counted the next day. Neutralization titers were determined to be the reciprocal of the last dilution of serum or purified material that reduced the number of plaques by 80% compared with the virus control wells.

### Viral challenge of mice

Female BALB/c mice (6 to 8 weeks of age) were used in all challenge experiments with maEBOV as described by Bray et. al [24]. Briefly, mice were injected with 1000 pfu or 100 pfu of maEBOV diluted in PBS. Virus challenge doses were delivered by IP injection in a volume of 0.2 ml. Interferon receptor knockout (IFNAR ^-/-^) mice (4 weeks of age) purchased from Jackson Labs (B6.129S2-Ifnar1tm1Agt/Mmjax) were challenged IP with 1000 pfu of wild type SUDV (Boniface) in a volume of 0.2 ml. All challenge studies involving the use of maEBOV and SUDV were performed at USAMRIID in animal biosafety level 4 laboratories. All animal research was conducted under an IACUC approved protocol in compliance with the Animal Welfare Act, PHS Policy, and other Federal statutes and regulations relating to animals and experiments involving animals. The protocol (AP-14-031) received approval by the U. S. Army Medical Research Institute of Infectious Diseases Institutional Animal Care and Use Committee on October 8, 2014. The facility where this research was conducted is accredited by the Association for Assessment and Accreditation of Laboratory Animal Care, International and adheres to principles stated in the Guide for the Care and Use of Laboratory Animals, National Research Council, 2011.

### Mouse antibody bioavailability study

The EBOV/SUDV pAbs were diluted in sterile PBS and delivered as single dose of 100 mg/kg in a total volume of 0.5ml via IP injection. Prior to and at various time points after administration of the EBOV/SUDV pAbs as described in the text, submandibular blood collection was performed and sera were isolated. The serum samples were then assayed by ELISA for total anti-EBOV and anti-SUDV IgG and by PsVNA for the presence of EBOV- and SUDV- neutralizing activity.

### Mouse passive transfer studies

For all studies, NS pAbs or EBOV/SUDV pAbs were diluted in sterile PBS to the specified dosing in a volume of 0.5 ml and delivered via IP injection. For the first challenge study with maEBOV, groups of 10 mice (~20 g each) were administered a single injection of negative control pAbs or EBOV/SUDV pAbs at a dose of 100 mg/kg. Control mice received NS pAbs one day prior to challenge while experimental mice received EBOV/SUDV pAbs at one day pre-challenge, or one or two days post challenge with 1000 pfu of virus. In the second maEBOV challenge study, groups of 10 mice (~20 g each) were challenged with 100 pfu of virus via IP injection. Control mice received the NS pAbs at a dose of 100mg/kg one day post challenge. Groups of experimental mice received a single injection of EBOV/SUDV pAbs at a dose of 100 mg/kg, 50 mg/kg, or 10 mg/kg starting one day post challenge. Additional groups of 10 mice received the same total doses listed above with half the dose administered one day after challenge and the second half dose administered two days after challenge. For the SUDV challenge, groups of 9 mice (~16 g each) received a single injection at a dose of 100 mg/kg of either NS pAbs one day prior to challenge or EBOV/SUDV pAbs one or two days post challenge with 1000 pfu of virus. For all challenge studies, mice were observed twice daily for clinical signs of disease for 21 days post-challenge and any animals found to be moribund were euthanized.

### Statistical analysis

GraphPad Prism software v6 for Windows (Graph, Inc.) was used to graph and conduct statistical analysis of all data. Briefly, two-way analysis of variance with Tukey’s *post hoc* tests was used to compare ELISA and PsVNA serum titers between time points within groups; two-way analysis of variance with Sidak’s *post hoc* tests was used to compare ELISA and PsVNA titers of purified samples between time points. Kaplan-Meier survival curve analysis and long-rank tests were performed for comparison of survival curves between groups. Probability (p) values <0.05 were considered statistically significant in all tests.
